# A context-based ABC model for literature-based discovery

**DOI:** 10.1371/journal.pone.0215313

**Published:** 2019-04-24

**Authors:** Yong Hwan Kim, Min Song

**Affiliations:** 1 Division of Humanities, CheongJu University, CheongJu, Korea; 2 Department of Library and Information Science, Yonsei University, Seoul, Korea; University of Sao Paulo, BRAZIL

## Abstract

**Background:**

In the literature-based discovery, considerable research has been done based on the ABC model developed by Swanson. ABC model hypothesizes that there is a meaningful relation between entity A extracted from document set 1 and entity C extracted from document set 2 through B entities that appear commonly in both document sets. The results of ABC model are relations among entity A, B, and C, which is referred as paths. A path allows for hypothesizing the relationship between entity A and entity C, or helps discover entity B as a new evidence for the relationship between entity A and entity C. The co-occurrence based approach of ABC model is a well-known approach to automatic hypothesis generation by creating various paths. However, the co-occurrence based ABC model has a limitation, in that biological context is not considered. It focuses only on matching of B entity which commonly appears in relation between two entities. Therefore, the paths extracted by the co-occurrence based ABC model tend to include a lot of irrelevant paths, meaning that expert verification is essential.

**Methods:**

In order to overcome this limitation of the co-occurrence based ABC model, we propose a context-based approach to connecting one entity relation to another, modifying the ABC model using biological contexts. In this study, we defined four biological context elements: cell, drug, disease, and organism. Based on these biological context, we propose two extended ABC models: a context-based ABC model and a context-assignment-based ABC model. In order to measure the performance of the both proposed models, we examined the relevance of the B entities between the well-known relations “APOE–MAPT” as well as “FUS–TARDBP”. Each relation means interaction between neurodegenerative disease associated with proteins. The interaction between APOE and MAPT is known to play a crucial role in Alzheimer’s disease as APOE affects tau-mediated neurodegeneration. It has been shown that mutation in FUS and TARDBP are associated with amyotrophic lateral sclerosis(ALS), a motor neuron disease by leading to neuronal cell death. Using these two relations, we compared both of proposed models to co-occurrence based ABC model.

**Results:**

The precision of B entities by co-occurrence based ABC model was 27.1% for “APOE–MAPT” and 22.1% for “FUS–TARDBP”, respectively. In context-based ABC model, precision of extracted B entities was 71.4% for “APOE–MAPT”, and 77.9% for “FUS–TARDBP”. Context-assignment based ABC model achieved 89% and 97.5% precision for the two relations, respectively. Both proposed models achieved a higher precision than co-occurrence-based ABC model.

## Introduction

With the development of modern biology, the number of publications in the biology literature has been increasing rapidly. As the size of the published literature increases, knowledge that is latent in the papers is also accumulated. Biomedical researchers increasingly have to search for the knowledge they want in a very large corpus. There has been considerable research into methods for automatically extracting knowledge from literature.

The ABC model of Swanson [1] has played a pioneering role in the literature-based discovery (LBD) field. The basic assumption of ABC model is that if entity B is associated with entity A in document set 1, and entity B and entity C in document set 2, the ABC model generates a hypothesis that entity A and entity C have a relation through entity B that appears commonly in both document sets. The result of ABC model is expressed as a path from entity A to entity C. This path allows us to hypothesize the relationship between entity A and entity C, or to help discover entity B as a new evidence for the relationship between entity A and entity C. ABC model has been a key model to discover new hypotheses through bio-literature mining.

Much research has been conducted based on this ABC model [2–9]. As there was a significant progress in LBD research based on Swanson’s ABC model, researchers become more interested in automatic methods such as Named Entity Recognition (NER) and Relation Extraction (RE) to extract knowledge in a large amount of scientific publications. Among various RE techniques, many LBD studies have been based on the co-occurrence-based RE approach [2–5]. Co-occurrence-based RE approach assumes that two entities have a relation if they are co-occurred in a sentence. Co-occurrence-based ABC model is a basic ABC model to apply the results of co-occurrence based RE approach to Swanson’s ABC model. For example, if "RSV" and "TP53" are co-occurred in a sentence and "TP53" has the relation of "Telomerase" in another sentence, “RSV—TP53” and “TP53—Telomerase" relations are extracted by the co-occurrence based RE approach. Applying these results to ABC model, it can be assumed that “RSV” is related to “Telomerase” through B entity, “TP53”. This co-occurrence based ABC model was applied to several recent studies [6, 9, 10].

However, LBD studies using the co-occurrence-based ABC model have a major limitation. The co-occurrence-based ABC model does not consider the context in which each entity relation appears. Since the co-occurrence-based ABC model is an inference model that identifies the relation between entity A and entity C through entity B, it does not focus on matching the contexts of entity relations, but focuses on matching to entity B which is common to both entity relations. Therefore, although a number of paths are constructed, it is difficult to understand many of the paths generated by the co-occurrence-based ABC model because of the existence of many erroneous connections between entity relations. For example, a connection may be deduced between an entity relation in mouse and an entity relation in humans. Eventually, the error rate of the paths elicited by the model becomes high, and evaluation by an expert is essential.

In order to overcome this limitation, we propose an approach using contexts. Contexts are defined as conditions or constraints that affect the relations between given entities. These may be locations, places or conditions where the entities interact. These concepts are the same as those of biological contexts in the biomedical field. Biological contexts include cells, tissues, or organisms in which the entities interact. It also includes environmental conditions such as drug-induced conditions, disease conditions, and air pollution. In biomedical experimental research, the biological context is rigorously defined and identified in an experimental design. A report on experimental results must include the biological context, because the results of biological experiments can vary extensively depending on the biological context, even in same experiment. Identifying the biological context in biomedical experimentation is important for the generation of new hypotheses and for generating data in support of hypotheses, because context establishes the conditions under which subsequent studies will be conducted. Therefore, it is necessary to consider biological context in order to generate a new hypothesis or to improve ABC model.

Using the concept of biological contexts, we propose two types of extension of ABC model, context-based ABC model and context-assignment-based ABC model. Context-based ABC model is an extension of ABC model that focuses on not only matching entity B but also matching contexts of entity relations which commonly occurred in both entity relations. Context-based ABC model allows us to observe more relevant paths from entity A to entity C. Context-assignment-based ABC model is a model complementing the limitation of Context-based ABC model. Because biological contexts are not included in every sentences, a small number of entity relations with biological contexts are extracted. Therefore, a small number of paths are generated by context-based ABC model. In order to overcome the limitation of context-based ABC model, we propose the method called Context-assignment based ABC model to assign biological contexts to each sentence in the abstract level.

ABC model is a methodology used to discover new knowledge from previously known knowledge. Therefore, it is difficult to verify the results of ABC model using public databases. For evaluation, we set entity A and entity C, where there is a well-known relation between two entities. We then tried to verify how meaningful B entities are extracted in the A-B-C path constructed by each ABC model. We compared the results of context-based ABC model and context-assignment-based ABC model to the results of co-occurrence-based ABC model. The results show that the precision of the B entities extracted using the context-based ABC model and context-assignment-based ABC model are higher than that of the B entities extracted using co-occurrence based ABC model.

This paper makes the following three contributions. First, we suggest context-based and context-assignment-based ABC models. The effectiveness of our proposed models is validated by comparing results with those of the co-occurrence-based model. Therefore, the B entities extracted by the context-based model can be considered reliable, and can be used to uncover mechanisms that have not previously been identified. Second, we propose the inclusion of the biological context of the relation between entities in ABC model. In the field of biomedical research, the use of other contexts except biological contexts is not important. In experiments and reports of their results, biological contexts play an important role in supporting experimental results or enabling new discoveries. Biological context reflects the actual research environment, so the ABC model taking into account biological context can provide more accurate results than the base model. Third, we propose a formula to calculate the similarity between two biological contexts. This formula helps to establish more accurate connections between entity relations by eliminating connections between entity relations which have biological contexts which are different or extraneous. It also makes it possible to construct networks including the context information. Therefore, a network can be constructed more accurately using the ABC model with biological contexts, so that more effective results can be expected when network-based analysis methods are applied to the network.

## Related work

Some studies have tried to overcome the limitations of the existing co-occurrence approach by using statistical techniques or thresholds. Hristovski et al. [11] proposed the LBD system, BITOLA, using semantic prediction. The system is combined with BioMedLEE, a type of NLP system, and SemRep to develop a model for RE. These authors applied their method to the identification of associations between Raynaud’s disease and fish oil, as studied by Swanson. In a study by Frijters at el. [12], CoPub, an LBD system to find new relations between biomedical concepts was developed and used to investigate relations between genes, therapeutic drugs, signaling pathways, and diseases. Lee et al. [13] investigated relations between biological processes and side effects using a drug as a B entity. They constructed a multilevel network by combining a drug-biological process network and a drug-side effect network.

Other studies tried to extract more accurate results by using verbs in the literature. Tsai et al. [14] used specific verbs for relation extraction. They developed a biomedical semantic role labeling system, BIOSMILE, to extract biomedical relations. In a study by Song et al. [7], a bio-literature mining tool, PKDE4J, was described. This tool is a dictionary-based system. It provides eight different types of entity annotation and relation annotation. The relation annotation is based on a biomedical verb dictionary. If two entities in one sentence have a relation with the main verb, and the main verb is included in the biomedical verb dictionary, that relation is annotated and extracted.

Another approach uses external information resources to overcome the limitations of the co-occurrence-based ABC model. Ijaz, Song, and Lee [15] proposed the MKE (Multi-Level Knowledge Emergence) model. The MKE model automatically extracts multidimensional biological entities from texts using ontologies such as UMLS and NLP (Natural Language Processing), and finds implicit relations between entities.

Another approach is network based. The ABC model is expanded to construct a network, and then network analysis algorithms are applied to the network. In a study by Seki et al. [16], an inference network was constructed and used to deduce relations between genes and diseases by combining Swanson’s approach and information retrieval technology.

Recently, some studies have tried to overcome the limitation of the co-occurrence-based ABC model by using the concept of context. However, the definition of contexts in the bio-literature mining field is not limited to biological contexts. Lee et al. [17] defined contexts as entities that co-occur with entity relations in the same journal paper abstract. In their method, if a similarity score based on the similarity between A–B’s context vector and B–C’s context vector exceeds a certain threshold value, entity relations are connected. A study by Cameron et al. [18] attempted to find the B entity by creating a graph. In their study, relations were extracted from all abstracts containing predefined A and C entities. A network was constructed from the extracted relations.

The concept of context has been used in research using the ABC model as well as in other research in bio-literature mining. In a study by Gerner et al. [19], gene-expression information and anatomical locations were extracted by applying a rule-based gene expression text miner to approximately 7,000 PubMed Central articles. They identified gene expression and its anatomical location or the cell line from which the data was extracted, using a dictionary-based approach. Neves et al. [20] extracted information about gene expression events with context from 2,376 articles using a dictionary-based approach. The context of their study was cell and anatomical location, similar to the context used by Gerner et al. [19]. The results were verified manually, and showed a precision of more than 50%. Yoon et al. [21] attempted to resolve conflicting relations in biological pathways using context. Poon’s [22] study used a distant supervision algorithm to extract biological pathways from PubMed. In their study, the MeSH term ‘cancer type’ was extracted as context and pathways depending on each context were extracted. They extracted about 1.5 million pathway interactions with about 25% accuracy from about 2.2 million PubMed abstracts.

Previous studies using biological context have identified different elements and varying numbers of contexts. In this study, we selected four elements based on the study by Yoon et al. [21]. However, a wide range of organisms are used in biological research. This study differs from Yoon et al. [21]’s study because we focused on hypothesis generation, specifically ABC model, and we used organism as an element of biological context.

## Materials and methods

### Overview of method

**Fig 1:**
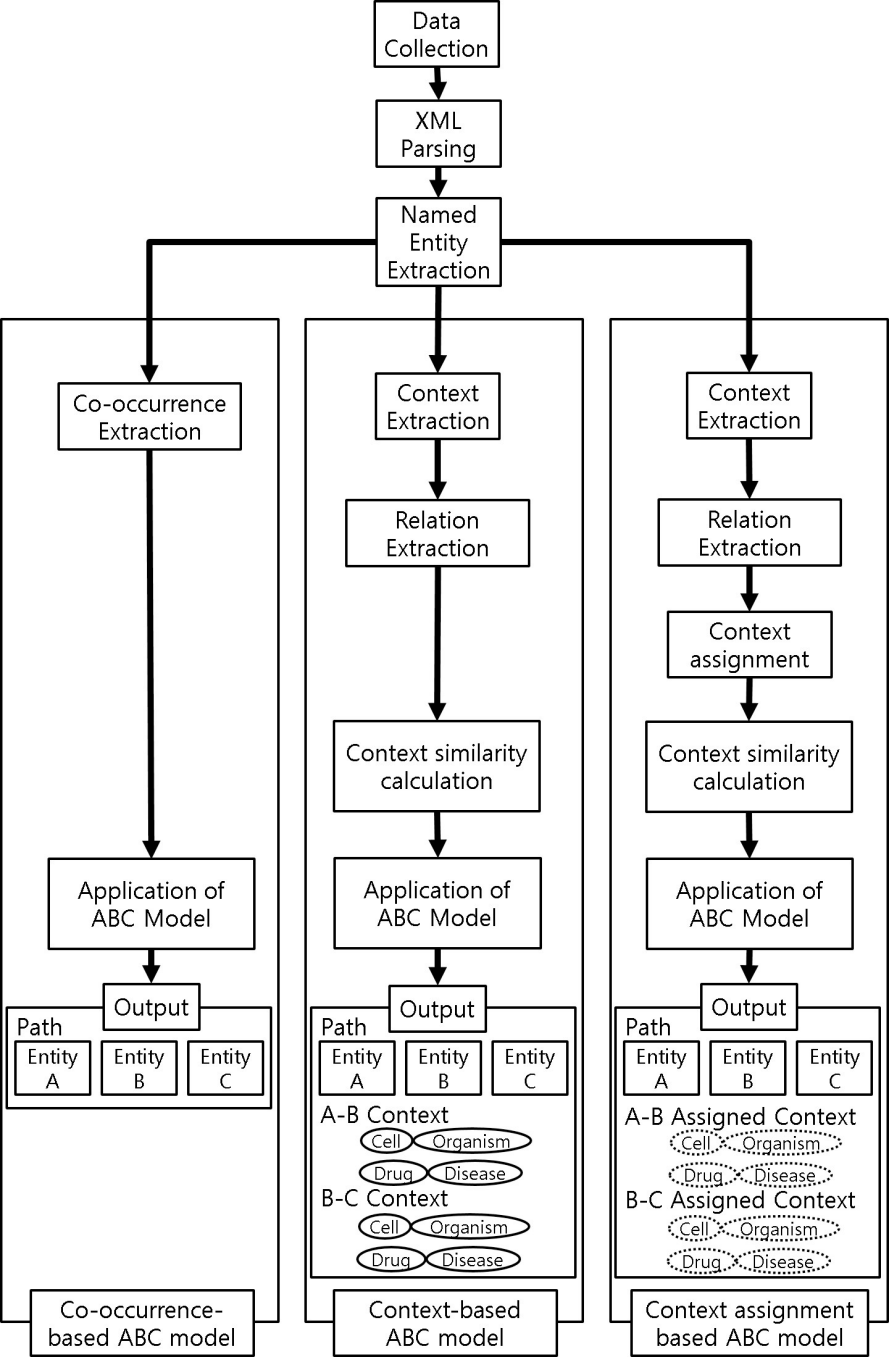
Outline of methodology.

Fig 1 outlines our methodology.

In this study, we extended PKDE4J [7]. We added two modules to the NER module provided in PKDE4J. One is a biological context extraction module. Using this module, we can extract biological context elements by determining if each entity extracted from a sentence is included in biological context. The other is context assignment module which is carried out after extraction of biological context elements. The module carries out to assign same biological context element to all sentences in an abstract. Finally, three results were derived using the extended PKDE4J. We then recorded the results of the context-based ABC model and the context-assignment-based ABC model. Context-based ABC model is applied based on the results of biological context extraction module whereas context-assignment-based ABC model is applied based on both context assignment module and biological context extraction module. The results of the co-occurrence based ABC model were used as a baseline. The performance of the proposed model was compared to the performance of the co-occurrence-based ABC model.

### Biological context extraction module

In this module, four biological context elements are extracted. As PKDE4J supports multi-type NER, it is possible to extract certain entity types as context elements. Therefore, PKDE4J is extended with a module determining the role of entities: context element or not.

**Fig 2:**
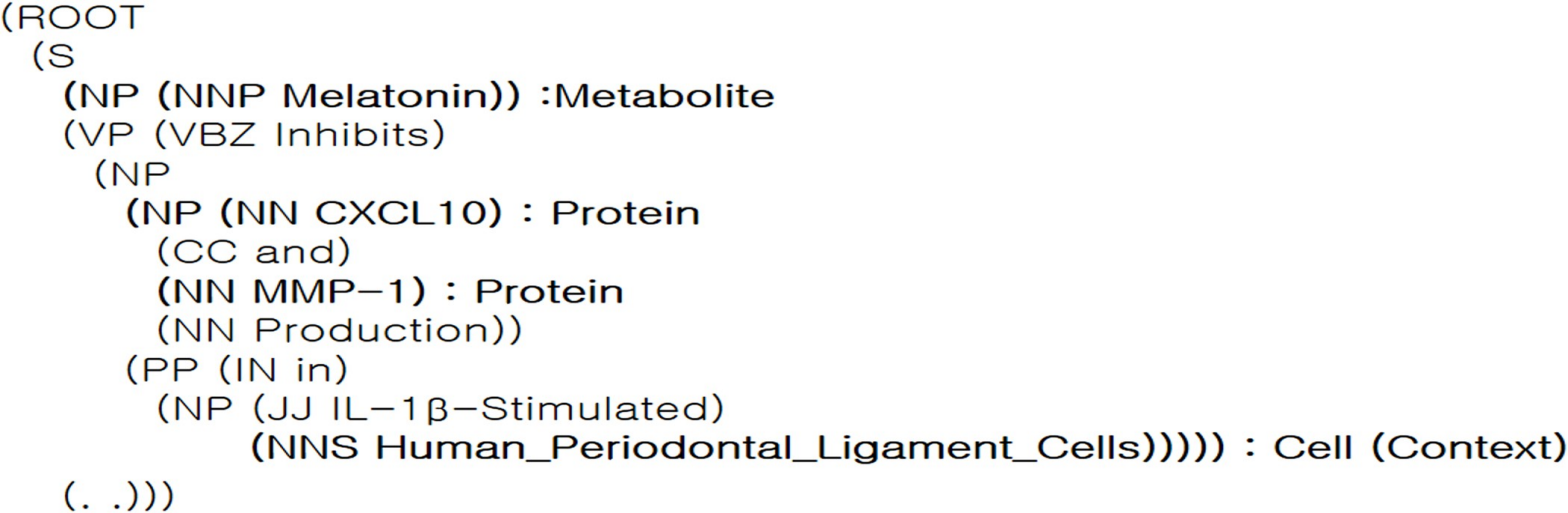
Example of tree parsing.

To determine if it is included in biological context elements, the pattern in which the biological context elements appear should be considered. The pattern of biological context elements is possible to consider using not only the pattern “Entity 1 is related to Entity 2 in the context element.” but also another pattern in which a context element is based on prepositional clauses or phrases and relative adverb clauses or phrases that present a condition. In the biological context extraction module, entities are extracted from the prepositional clauses or phrases, or relative adverb clauses as context candidates using tree parsing of the Stanford Core NLP [23]. And then if the context candidate is one of the entity types cell, disease, drug or organism, the entity is extracted as biological context element. Fig 2 is an example of a tree parsing provided by the Stanford NLP. Using this syntax tree, we can find prepositional phrases or clauses and relative adverb phrases or clauses that affect an entire sentence, and we can extract context elements from those phrases and clauses. In Fig 2, the “PP,” preposition phrase, consists of “in,” “IL-1β–stimulated,” and the phrase “human periodontal ligament cells.” In this case, an entity “human periodontal ligament cell” is extracted as a cell element of the biological context. Generally, it is difficult to find a sentence including all four context elements. Therefore, elements which have not been identified are presented as “NoData” and that element is excluded from the calculation of context similarity.

The RE results of the original PKDE4J consist of PMID, Verb, Entity 1, Entity 2, Negation and Tense per sentence. PMID is the identifier of a PubMed articles. Verb is the main verb that connects between the left entity and the right entity. Of two entities, entity 1 is a leading one and entity 2 is a trailing one in the sentence. Negation is either POSITIVE or NEGATIVE. It means that the relationship between two entities is either positive or negative. Tense is either PASSIVE or ACTIVE. ACTIVE means that the direction of the effect is from entity 1 to entity 2 whereas PASSIVE is inversed. The RE result of extended PKDE4J provides Entity 1 official symbol, Entity 2 official symbol, and context elements. A large portion entities have various alias since researchers named the same entity by synonymous terms. In order to integrate various alias, we used an official symbol from dictionaries used in PKDE4J, and we added it to the results of the biological extraction module. Table 1 shows an example of extracted entity relations from an article with PMID 23829269[24]. The original sentence of Sentence ID 1 is “Hsf-1 affects podocyte markers NPHS1, NPHS2 and WT1 in a transgenic mouse model of TTRVal30Met-related amyloidosis.” And Sentence ID 8 is “Nephrin, podocin and WT1 gene expression levels were unaffected by the Hsf-1 carrier status.” Of them, entity relation with the sentence ID 8 has no context elements. Due to that, the entity relation is excluded in context-based ABC model.

**Table 1:** Example of entity relations extracted from an abstract using the biological context extraction module.

PMID	Sentence_ID	Entity 1	Entity 1 Official Symbol	Entity 2	Entity 2 Official Symbol
23829269	1	hsf-1	nr5a1	nphs1	nphs1
23829269	8	wt1	wt1	hsf-1	nr5a1
		**Negation**	**Voice**	**Verb**	**Re_Type**
23829269	1	POSITIVE	ACTIVE	affect	AFFECTS
23829269	8	POSITIVE	ACTIVE	unaffected	AFFECTS
		**CONTEXT**			
		**CELL**	**DRUG**	**DISEASE**	**ORGANISM**
23829269	1	NoData	NoData	amyloidosis	transgenic mouse
23829269	8	NoData	NoData	NoData	NoData

### Context assignment

Some sentences do not contain any biological context. If both entity relations and biological contexts are extracted in a sentence, it is clear that the relation is affected by the biological contexts. However, biological contexts are not included in every sentence, as every sentence does not contain the entity relation. Therefore, a small number of entity relations with biological contexts are extracted entity relations without biological contexts are discarded. For example, in Table 1, entity relation with sentence id 1has the relation hsf-1 –nphs1 and the biological contexts transgenic mouse (organism) and amyloidosis (disease) were extracted. In entity relation with sentence id 8, only the hsf-1 –wt1 entity relation was extracted, without biological contexts. In the context-based ABC model, only the hsf-1 –nphs1 entity relation is used, while the hsf-1 –wt1 entity relation was discarded because of the absence of contexts. Therefore, in order to reduce the overlooking of significant amounts of knowledge, it is necessary to assign biological contexts to entity relations without identified biological contexts.

In this study, we propose a method to assign biological context to each sentence in an abstract. Suppose that the entity relation “A—B” and the biological context are extracted from one sentence in an abstract, and only the entity relation “C—D” is extracted from another sentence in the same abstract. In this case, the biological context of “A—B” is assigned to “C—D”. The basic assumption of this context assignment is that one extracted biological context with an entity relation has a high possibility of impacting another entity relation extracted from the same abstract. We applied this assumption to the ABC model. Table 2 shows an example of the way in which biological context is assigned to entity relations without a biological context in same example from Table 1. The biological contexts transgenic mouse and amyloidosis were extracted as shown in Table 1. The combination of biological contexts is organized as transgenic mouse (organism) and amyloidosis (disease). These biological contexts are assigned to the hsf-1 –wt1 entity relation, as shown in Table 2. If the extracted biological context elements are different, the biological context consists of a combination of all extracted elements. If the extracted biological contexts are assigned only to entity relations without biological context, the influence of the entity relation including the actual biological context may be lowered. Therefore, we proposed that all entity relations extracted from the same abstract have the same biological contexts in the context-assignment module.

**Table 2:** Example of entity relations extracted by Context-assignment-based ABC model.

PMID	Sentence_ID	Entity 1	Entity 1 Official Symbol	Entity 2	Entity 2 Official Symbol
23829269	1	hsf-1	nr5a1	nphs1	nphs1
23829269	8	wt1	wt1	hsf-1	nr5a1
		**Negation**	**Voice**	**Verb**	**Re_Type**
23829269	1	POSITIVE	ACTIVE	affect	AFFECTS
23829269	8	POSITIVE	ACTIVE	unaffected	AFFECTS
		**CONTEXT**			
		**CELL**	**DRUG**	**DISEASE**	**ORGANISM**
23829269	1	NoData	NoData	amyloidosis	transgenic mouse
23829269	8	NoData	NoData	amyloidosis	transgenic mouse

### Measurement of context similarity

**Fig 3:**
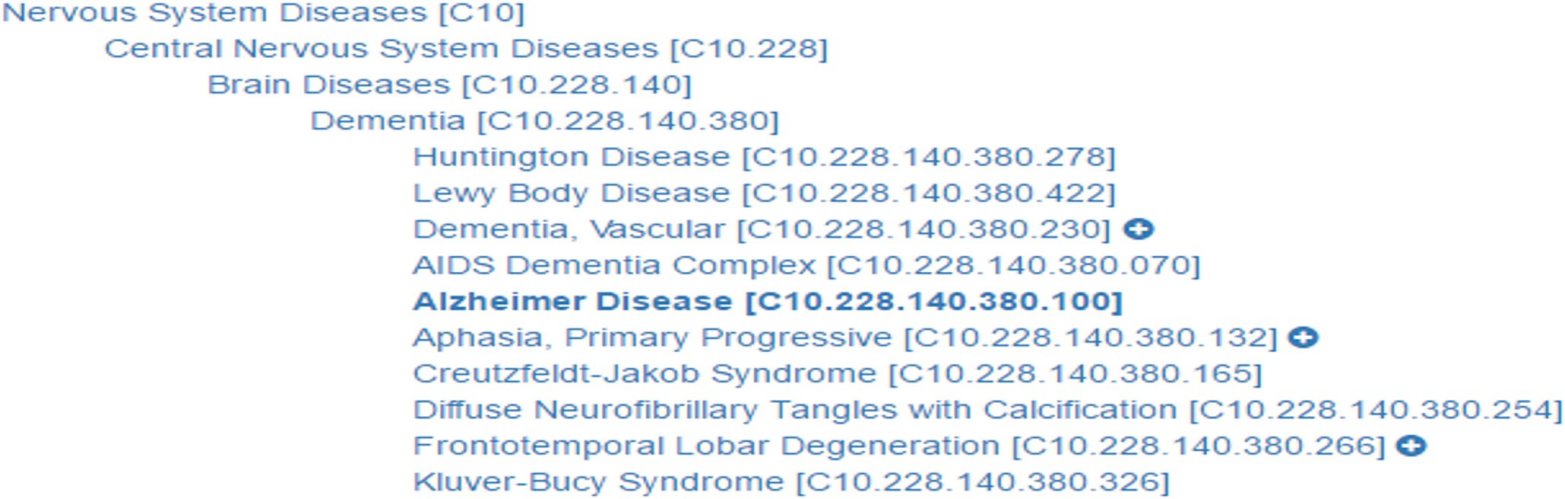
Example of MeSH hierarchical structure of Alzheimer’s disease.

When the context-based ABC model is applied, it is necessary to measure a similarity score between biological contexts, in order to connect one entity relation with another. In this study, each element was set as an independent element, because it is difficult to determine which element has more importance. The similarity measurement of each element was calculated using relatedness between elements in a hierarchical structure. Each element includes hierarchical information. The hierarchical information was collected from public databases. The structure of the hierarchy of cells and diseases comes from MeSH. For drugs, the hierarchical structure was provided by DrugBank [25]. Organisms were collected using the KEGG organism database [26]. Fig 3 shows an example of the hierarchy of “Alzheimer’s disease” in MeSH. In Fig 3, diseases at a higher level include lower level diseases, and the diseases at a low level with the same upper level disease are known as diseases of similar symptoms. “Huntington disease” and “Alzheimer’s disease” is sub-disease of “Dementia”, both are known as similar diseases. Therefore, using this hierarchy we can estimate similarity between biological context elements.

Because each entity has a hierarchical structure, it is possible to calculate the distance between entities. The calculated distance is normalized with reference to the maximum distance in the hierarchical structure. The maximum distance of each context element is different for each element type. The maximum distances we identified were 30 for cells, 14 for drugs, 26 for diseases, and 12 for organisms. We developed a formula to convert from normalized distance to similarity. The following is the similarity formula applied to each element of the biological context:

$$Similarity\;of\;element=1-\frac{Distance\;Between\;element1\;and\;element\;2}{Maximum\;Distance}$$ (1)

Finally, the similarity score in biological context was calculated using the following formula:

$$Context\;Similarity=\frac{Cell\;Similarity+Drug\;Similarity+Disease\;Similiarity+Organism\;Similarity}{\sum \sqrt{Number\;of\;element\;in\;relation\;1}\sum \sqrt{Number\;of\;element\;in\;relation\;2}}$$ (2)

**Fig 4:**
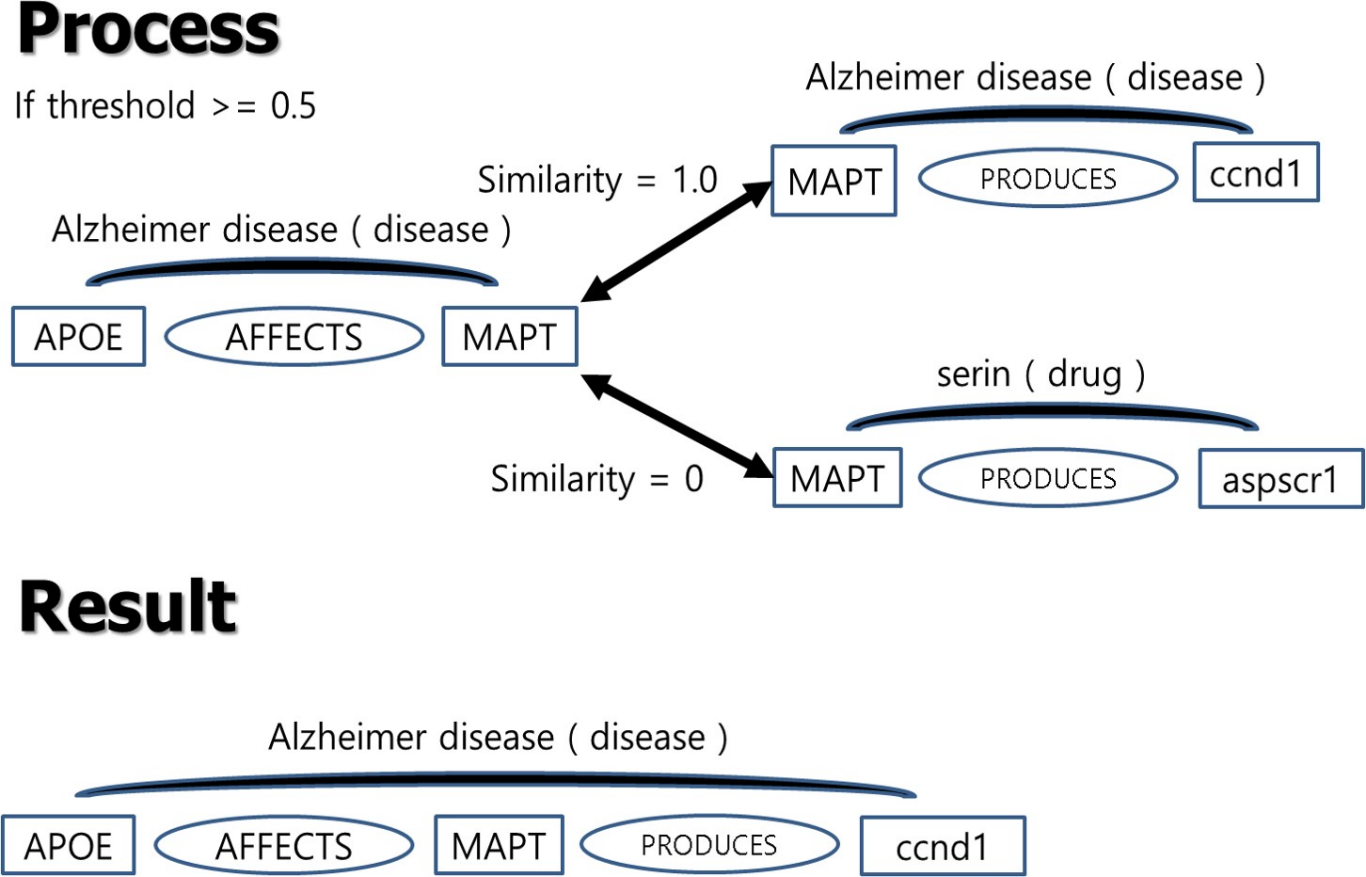
Example of method to connect entity relations.

This formula is a modification of the cosine similarity formula. We summed all of the similarity scores calculated from each context element, and then normalized the similarity value by the number of extracted biological context elements. Not all entity relations have the same number of biological context elements, and not all biological context elements are extracted together. Therefore, the number of biological context elements is used for the similarity score. Using this formula, we propose the method used to connect entity relations, depending on the biological context similarity score. If the calculated similarity score is more than the threshold, one entity relation is connected with another. If not, the two entity relations are disconnected. In Fig 4, “APOE–MAPT” and “MAPT–ccnd1” have the same biological context, Alzheimer’s disease, with similarity score 1.0 which is over the threshold. On the other hand, “APOE–MAPT” and “MAPT–aspscr1” have different biological contexts. Its biological context similarity is 0 which is below the threshold. Eventually, “APOE–MAPT” and “MAPT–ccnd1” are connected, while “APOE- MAPT” and “MAPT–aspscr1” are disconnected.

### Context-based and context-assignment-based ABC model

Using extended PKDE4J, we proposed two models, context-based ABC model and context-assignment-based ABC model. Context-based ABC model is a model based on the biological context extraction module and a context similarity formula. It considers the matching of the context of each entity relations as well as the matching of B entity, which is the core of the existing ABC model. While many irrelevant relations appeared in the results by the existing model because it only considers entity B, the proposed model has an advantage that it provides more suitable results by removing irrelevant relations from the results of the existing ABC model. This overcomes the limitation of ABC model. However, in this model, entity relations without biological contexts are excluded. This means that a significant amount of knowledge in the literature is filtered. Even though the precision of context-based ABC model is higher than ABC model’s because of its use of biological contexts, it results in weakening the performance of recall.

Context-assignment-based ABC model uses both biological context extraction, context assignment modules, and a context similarity formula. It aims to solve the problem of context-based ABC model excluding a large amount of relations. With the application of this model, we can sustain a large amount of relations for path analysis, and one of our experiment results showed that recall is increased in this model whereas recall is lowered in context-based ABC model. It shows a possibility to overcome low performance of recall in context-based ABC model. However, since this model estimates the context in which entity relation appears, it has a limitation that it generates more irrelevant relations than ones generated by context-based ABC model.

## Results

### Data collection

Because of an aging society and the increasing complexity of societies, interests in mental disorders, and in particular, neurodegenerative diseases, have increased. Recently the concern about dementia, which is a type of neurodegenerative disease, has grown. Neurodegenerative diseases include Parkinson’s disease, Alzheimer’s disease, Huntington’s disease, amyotrophic lateral sclerosis, and dementia. In this study, we applied our proposed method to the literature concerning these neurodegenerative diseases.

We collected 275,318 XML records involving neurodegenerative disease from PubMed on March, 2017. Using SAX parsing by manual JAVA programming, we collected PubMed ID, title, abstract, and year of publication from the XML. Articles without abstracts were removed, and finally, 214,621 articles were used for the experiment.

### Evaluation

We propose a method to make new and highly accurate discovery using biological contexts, by applying the proposed ABC model. However, the ABC model is a methodology used to discover new knowledge from previously known knowledge or to create new hypotheses. Therefore, it is difficult to find a validated corpus for this methodology. Public databases are also not suitable for the evaluation of the B entities or the entity relations generated through the ABC model. Smalheiser and Torvik [27] also mentioned the difficulty of verifying the ABC model. Therefore, it is natural that results are verified by experts in other studies using the ABC model. Many of the previous studies have used expert evaluations to validate the ABC model [22, 28, 29].

In this study, the results of applying the ABC model were verified by experts. We extracted two entities known to be related and evaluated the validity of the B entity between them. The relations, “APOE–MAPT” and “FUS–TARDBP”, which have been verified using BioGrid [30] were selected for evaluation. Each relation means interaction between neurodegenerative diseases associated with proteins. APOE is a fat binding protein that is involved in the metabolism of fat and other lipoproteins. MAPT also known as Tau plays a key role in modulating the stability of axonal microtubules. The interaction between APOE and MAPT is known to play a crucial role in Alzheimer’s disease as APOE affects tau-mediated neurodegeneration [31]. FUS is a RNA-binding protein which is known to play a role in transcriptional activation and mediates DNA repair. TARDBP is a DNA binding protein but also has been found to bind to RNA. TARDBP plays multiple roles as a transcriptional repressor. It has been shown that mutation in FUS and TARDBP are related to amyotrophic lateral sclerosis(ALS), a motor neuron disease by leading to neuronal cell death [32]. In order to compare the validity of the B entities generated by the context-based ABC model and the context-assignment-based ABC model using these two entity relations, the B entities extracted by the co-occurrence-based ABC model were verified using the same methodology.

The validation is carried out by three experts using the KEGG pathway database [33], Entrez Gene [34], and scientific publications. The KEGG pathway database is one of the databases provided by Kyoto Encyclopedia of Gene and Genomes (KEGG). It provides pathway maps representing various functions of the cell and the organism. It also includes molecular interaction and gene-pathway association. Entrez Gene is a gene database provided by NCBI. It provides various gene or genome information including gene type, genomic context, gene expression, associated gene ontology, SNP, phenotype, pathway, and visualized gene information. Using these databases, experts can verify relevance of B entities. The others that were not confirmed by these databases were verified by finding evidence from scientific publications.

In the case of disagreement between the experts, the decision was made based on the majority of the opinions. All opinions by the experts are in S1 and S2 Tables. In addition, among various evaluation measurement scores, we focused on precision. In the biomedical field, high precision is typically more important than high recall. Therefore, in this study, all performance evaluations are presented as precision values.

### The results of relation extraction

Three results of the RE were extracted from 214,621 PubMed records: the results of the co-occurrence-based ABC model, the context-based ABC model, and the context-assignment-based ABC model. Table 3 shows the number of entity relations extracted using each method.

**Table 3:** The results of relation extraction.

Co-occurrence based ABC model	Context based ABC model	Context-assignment based ABC model
53,850	13,640	33,448

The co-occurrence based ABC model generated the largest number of entity relations because entity relations are extracted based on simple co-occurrence approach, and all extracted entity relations are not filtered. The context-based ABC model extracts the smallest number of entity relations, because a lot of entity relations without biological contexts are discarded. And it may lead a small number of paths. The context-assignment-based ABC model produces some entity relations that do not have biological context. Therefore, more entity relations than those from the context-based ABC model were generated.

Tables 4 and 5 are examples of entity relations extracted from the context-based and co-occurrence-based ABC models. Given the sentence, "Hsf-1 affects podocyte markers NPHS1, NPHS2 and WT1 in a transgenic mouse model of TTRVal30Met-related amyloidosis," three entity relations, with relation type and biological context, were extracted by the context-based ABC model (Table 4).

**Table 4:** An example of entity relations in context-based ABC model.

PMID	Sentence_ID	Entity 1	Entity 1 Official Symbol	Entity 2	Entity 2 Official Symbol
23829269	1	hsf-1	nr5a1	nphs1	nphs1
23829269	1	hsf-1	nr5a1	nphs2	nphs2
23829269	1	hsf-1	nr5a1	wt1	wt1
		**Negation**	**Voice**	**Verb**	**Re_Type**
23829269	1	POSITIVE	ACTIVE	Affect	AFFECTS
23829269	1	POSITIVE	ACTIVE	Affect	AFFECTS
23829269	1	POSITIVE	ACTIVE	Affect	AFFECTS
		**CONTEXT**			
		**CELL**	**DRUG**	**DISEASE**	**ORGANISM**
23829269	1	NoData	NoData	Amyloidosis	transgenic mouse
23829269	1	NoData	NoData	Amyloidosis	transgenic mouse
23829269	1	NoData	NoData	Amyloidosis	transgenic mouse

**Table 5:** An example of entity relations in co-occurrence-based ABC model.

PMID	Sentence_ID	Entity 1	Entity 1 Official Symbol	Entity 2	Entity 2 Official Symbol
23829269	1	hsf-1	nr5a1	nphs1	nphs1
23829269	1	hsf-1	nr5a1	nphs2	nphs2
23829269	1	hsf-1	nr5a1	wt1	wt1
23829269	1	nphs1	nphs1	nphs2	nphs2
23829269	1	nphs1	nphs1	wt1	wt1
23829269	1	nphs2	nphs2	wt1	wt1

Six entity relations were extracted by the co-occurrence-based ABC method (Table 5). Four genes, Hsf-1, nphs1, nphs2, and wt1, are related to each other in the model. However, in fact only hsf-1 has a relationship to the other three genes, and it is hard to find actual relations in the co-occurrence-based ABC model because of characteristics of the model.

The difference in the number and associated information of entity relations results from the module added to PKDE4J. In the context-based ABC model, we used the NER, biological context extraction, and RE modules with PKDE4J. The RE module extracts entity relations using several strategies [7]. Therefore, more accurate entity relations can be extracted. On the other hand, in the co-occurrence-based ABC model, we used only NER modules.

### Verification in application of ABC model

Table 6 shows an example of the results from context-based ABC model applying to “APOE–MAPT”. Each low in Table 6 shows paths from APOE to MAPT with biological contexts. Some paths have different biological contexts, even if those paths are identical. In this study, we also carried out comparison based on biological context similarity. Namely, A-B context of a path can be different from B-C context. With this result, we verified B entities between “APOE” and “MAPT” after deduplication. Although Table 6 shows nineteen paths, the number of B entities is seven after deduplication. Three experts verified seven B entities in the case of “APOE–MAPT”. In addition, Table 6 includes the results by Context-assignment-based ABC model.

**Table 6:** The example of results from context-based ABC model (APOE–MAPT).

Path			A-B Context				B-C Context			
Entity A	Entity B	Entity C	Cell	Drug	Disease	Organ-ism	Cell	Drug	Disease	Organ-ism
Apoe	ins	Mapt	·	Glucose	·	·	·	Glucose	·	·
Apoe	c9orf72	Mapt	·	·	frontotemporal dementia	·	·	·	frontotemporal dementia	·
Apoe	snca	Mapt	·	·	parkinson disease	·	·	·	parkinson disease	·
Apoe	snca	Mapt	·	·	parkinson disease	·	·	·	parkinson disease	·
Apoe	phgdh	Mapt	·	·	Dementia	·	·	·	Dementia	·
Apoe	snca	Mapt	·	·	supranuclear palsy, progressive	·	·	·	supranuclear palsy, progressive	·
Apoe	c9orf72	Mapt	·	·	frontotemporal dementia	·	·	·	frontotemporal dementia	·
Apoe	snca	Mapt	·	·	parkinson disease	·	·	·	parkinson disease	·
Apoe	bche	Mapt	·	glucose	·	·	·	glucose	·	·
Apoe	c9orf72	Mapt	·	·	frontotemporal dementia	·	·	·	frontotemporal dementia	·
Apoe	src	Mapt	·	l-tyrosine	·	·	·	l-tyrosine	·	·
Apoe	phgdh	Mapt	·	·	dementia	·	·	·	dementia	·
Apoe	snca	Mapt	·	·	multiple system atrophy	·	·	·	multiple system atrophy	·
Apoe	src	Mapt	·	l-tyrosine	·	·	·	l-tyrosine	·	·
Apoe	ins	Mapt	·	glucose	·	·	·	glucose	·	·
Apoe	ins	Mapt	·	glucose	·	·	·	glucose	·	·
Apoe	src	Mapt	·	l-tyrosine	·	·	·	l-tyrosine	·	·
Apoe	bche	Mapt	·	Glucose	·	·	·	Glucose	·	·
Apoe	mcidas	Mapt	·	·	Dementia	·	·	·	dementia	·

### Verification of B entities between APOE and MAPT

S1 Table shows the three expert opinions and evidence for the B entities extracted by the co-occurrence-based ABC model. The evidence followed the majority opinion. A total of 166 B entities appeared after removal of duplicates, and 45 genes were associated with APOE and MAPT. It has a precision of 27.1%. The threshold applied to the context similarity score in the context-based ABC model was 1, which reflects exact matching. A single path has two entity relations with the same biological context. Nineteen paths were extracted by the context-based ABC model as shown Table 6. The number of B entities was seven after removal of duplicates. Table 7 depicts the validity of the seven B entities extracted by the context-based ABC model.

**Table 7:** Verification of B entities using the context-based ABC model (APOE—MAPT).

	B entity	Verifi- cation	Evidence
1	snca	X	1) SNCA as well as ApoE has been associated with cognitive decline in neurodegenerative disease [35]. 2) SNCA and mapt have no direct relation.
2	phgdh	O	1) ApoE and phgdh have interaction [36] 2) Serine (phgdh is involved in serine synthesis) mutations in mapt increases mapt aggregation [37]
3	ins	O	1) ApoE4 reduces brain insulin (ins) [38] 2) Insulin dysfunction induces in vivo mapt hyperphosphorylation [39].
4	bche	O	1) ApoE and bche functions as modulators of cerebral amyloid deposition [40]. 2) BChE-K(BChE varient) is associated with reduced mapt phosphorylation [41].
5	c9orf72	O	1) c9orf72 is a stronger determinant than Apoe of cognitive impairment in ALS [42]. 2) Mutations in c9orf72 and mapt are found in familial frontotemporal dementia [43].
6	mcidas	X	1) ApoE is required for cell cycle regulation (mcidas plays a role in mitotic cell cycle progression by promoting cell cycle exit) [44]. 2) No direct evidence of interaction between mcidas and mapt.
7	src	O	1) ApoE binding stimulates intracellular activation of Src[45]. 2) SRC family kinases phosphorylates mapt [46].

**Fig 5:**
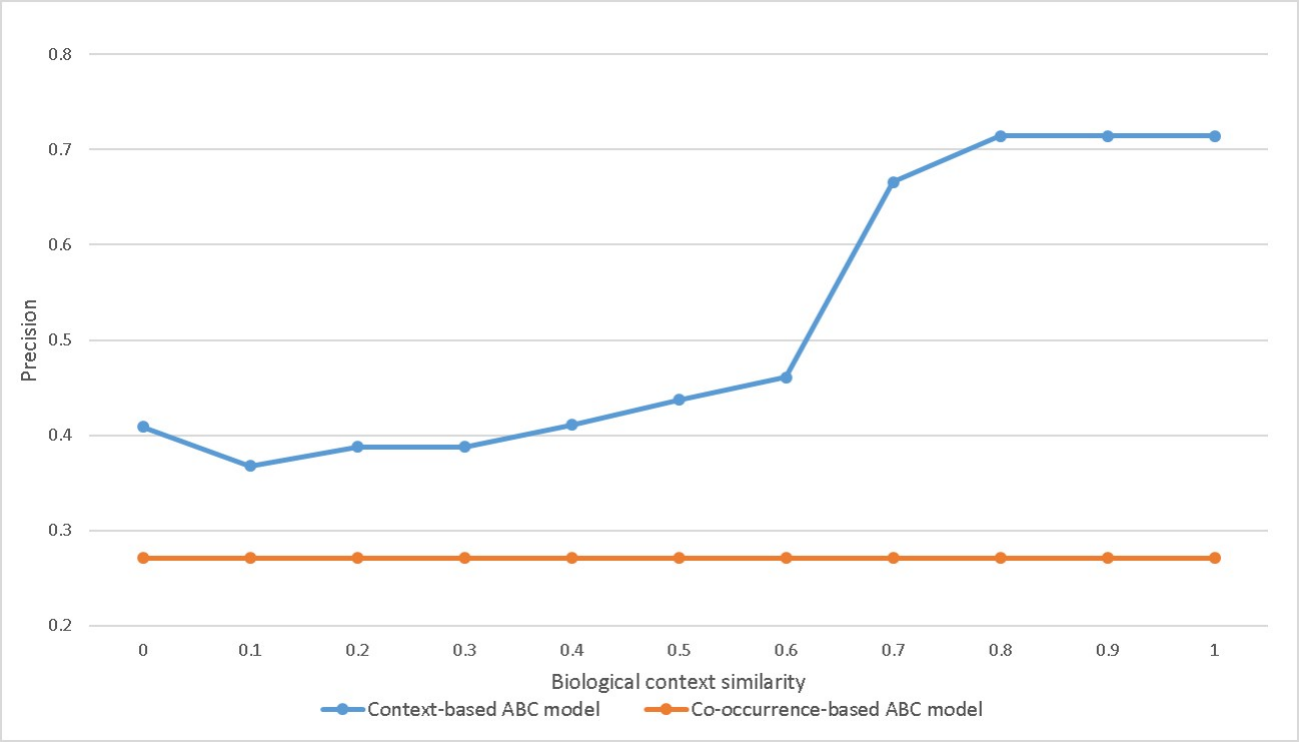
Change of precision according to biological context similarity threshold.

Five genes, aside from mcidas and snca, play roles in a relevant B entity. A literature search validates the relevance of the B entity. In the case of mcidas, the relation between mcidas and APOE has been established in the literature, but there is no evidence of a relation between mcidas and MAPT. The relation between snca and APOE is supported in the literature, but there is no evidence of a relation between snca and MAPT. Except for these, all of the others were judged to be relevant B entities. The model performed with 71.4% precision. Here, B entities generated by the co-occurrence-based ABC model produced a number of false positives and had low precision. Although the number of paths generated by the context-based ABC model was low, a number of the B entities were relevant, and the model had high precision. However, recall was low due to the small number of paths. The recall was 11.1%, assuming that the 45 genes from the co-occurrence based ABC model were all suitable B entities. Fig 5 shows the precision for the extracted B entities based on the threshold of similarity score between the biological contexts. This result is a comparison of context-based ABC model and co-occurrence-based ABC model. The context-based ABC model showed higher precision, depending upon the threshold, with the highest precision at 71.4% when the threshold is above 0.8. The context-based ABC model's precision was higher than the co-occurrence-based ABC model's precision for all threshold values.

The context-assignment-based ABC model was also verified at a threshold value 1. In total 86 paths were constructed, and 20 B entities were extracted after deduplication. Fourteen of the 20 B entities were relevant B entities. The model's precision was 70%, which was 1% lower than the context-based ABC model's performance, but the model was more effective because more B entities could be extracted, and it had a higher recall value than the context-based ABC model. Table 8 shows the results of verifying the extracted B entities in the context-assignment-based ABC model.

**Table 8:** Verification of B entities from the context-assignment-based ABC model (APOE-MAPT).

	B entity	Verifi- cation	Evidence
1	app	O	1) ApoE elevates the transcription of APP. 2) APP metabolism regulates mapt proteostasis[47].
2	bace1	O	1) APOE and bace1 levels show relation 2) Mapt hyperphosphorylation is related with increased bace1
3	bche	O	1) ApoE and bche functions as modulators of cerebral amyloid deposition [40]. 2) BChE-K(BChE varient) is associated with reduced mapt phosphorylation [41].
4	c9orf72	O	1) c9orf72 is a stronger determinant than Apoe of cognitive impairment in ALS [42]. 2) Mutations in c9orf72 and mapt are found in familial frontotemporal dementia [43].
5	clu	X	ApoE, clu, and mapt have no direct relation.
6	cr1	O	CR1 interacts with APOE and affects mapt.
7	ctsd	O	1) CTSD and APOE have been associated with cognitive ability 2) CTSD is associated with degrading mapt
8	cyp46a1	O	CYP46A1 may interact with APOE to influence phospho-mapt protein
9	gfap	O	GFAP-apoE is associated with increased phosphorylation of mapt
10	grn	X	APOE and grn are in independent pathway having no relation
11	hfe	O	1) HFE mutations correlate with APOE 2) HFE increases mapt phosphorylation
12	ins	O	1) ApoE4 reduces brain insulin (ins) [38] 2) Insulin dysfunction induces in vivo mapt hyperphosphorylation [39].
13	mcidas	X	1) ApoE is required for cell cycle regulation (mcidas plays a role in mitotic cell cycle progression by promoting cell cycle exit) [45]. 2) No direct evidence of interaction between mcidas and mapt.
14	phgdh	O	1) ApoE and phgdh have interaction [36] 2) Serine (phgdh is involved in serine synthesis) mutations in mapt increases mapt aggregation [37]
15	picalm	O	1) PICALM and APOE is associated 2) PICALM modulates mapt accumulation [48].
16	prnp	X	Pnrp and fus are in independent path ways not affecting each other
17	rcan1	O	ApoE genotype shows higher levels of RCAN1 and phospho-ta
18	sars2	X	APOE and sars2 are in independent pathway having no relation
19	snca	X	1) SNCA as well as ApoE has been associated with cognitive decline in neurodegenerative disease [35]. 2) SNCA, mapt have no direct relation.
20	tardbp	O	1) APOE formed complex with TARDBP 2) TARDBP and mapt protein levels are related

**Fig 6:**
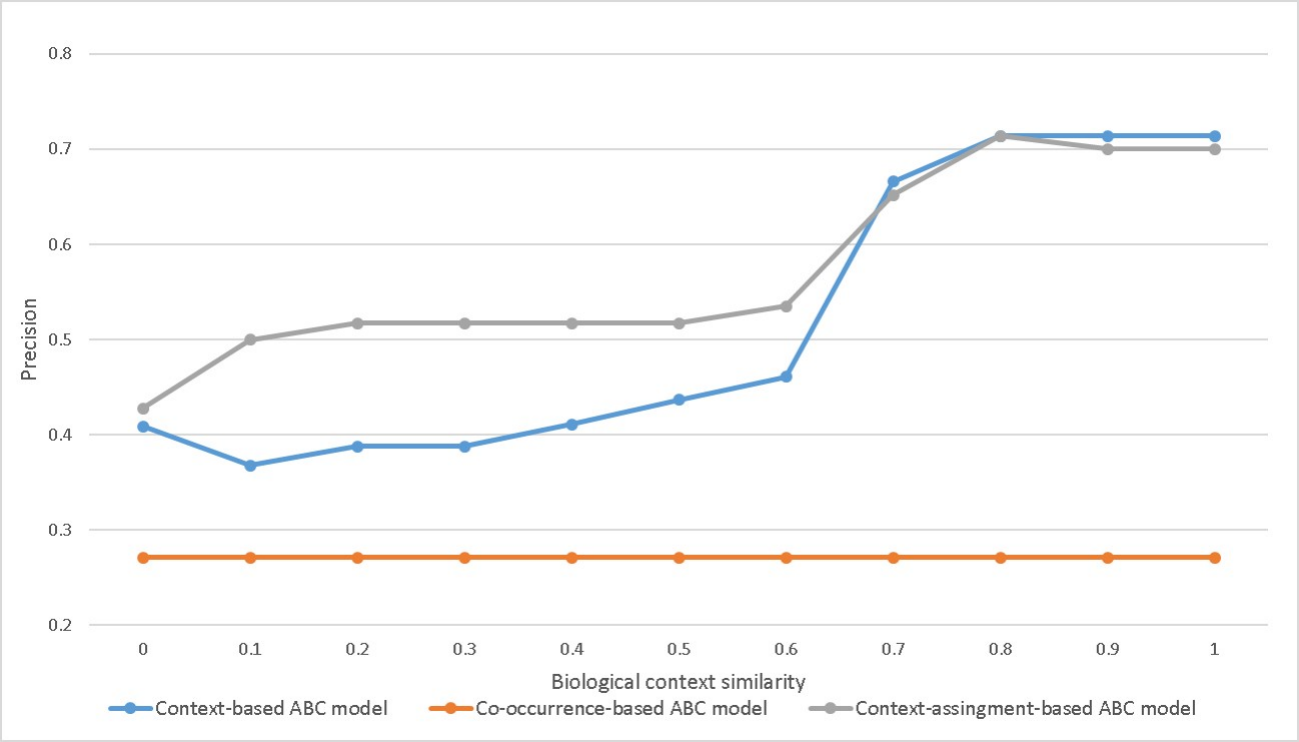
Comparison of Context-based, context-assignment based and co-occurrence based ABC model (APOE—MAPT).

In addition, the context-assignment-based ABC model was compared with the co-occurrence-based and context-based ABC models. Fig 6 shows the comparison between the context-based ABC model, the context-assignment-based ABC model, and the co-occurrence-based ABC model depending upon threshold. The context-assignment-based ABC model's performance was slightly lower than that of the context-based ABC model when the threshold was more than 0.7. The context-assignment-based ABC model had the highest performance when the threshold was less than 0.7. The context-based and context-assignment-based ABC models also had higher performance than the co-occurrence-based ABC model. The results show that the use of biological contexts is effective in the ABC model in connecting one entity relation to another and finding relevant B entities.

### Verification of B entities between FUS and TARDBP

Additional verification was carried out on B entities between “FUS” and “TARDBP”. Table 9 shows the verification of the context-based ABC model on extracted B entities. In Table 9, among nine extracted genes, all the genes except Sod1 played a relevant role between FUS and TARDBP, with 88.9% precision. Sod1 had no relation to FUS and TARDBP and acts independently of them.

**Table 9:** Verification of B entities from context-based ABC model (FUS-TARDBP).

	B entity	Verifi- cation	Evidence
1	sod1	X	Sod1 acts independently of fus and tardbp [49].
2	gli3	O	1) Fus is associated with ALS while, gli3 is associated with ALS through the shh pathway [50,51]. 2) Sonic hedgehog signaling in which gli3 participates and notch signaling can cooperate to regulate neurogenic divisions. Notch signaling may rescue tardbp [52,53].
3	c9orf72	O	1) Fus is associated with endosomal trafficking [54]. 2) Tardbp loss of function inhibits endosomal trafficking, c9orf72 regulates endosomal trafficking [55, 56].
4	vapb	O	1) Fus disrupts the vapb interactions to other signaling proteins [57]. 2) Mutations in vapb have already been shown to cause cytoplasmic transactive response tardbp accumulations [58].
5	mapt	O	1) Fus alternatively splices mapt [59]. 2) Tardbp is a component of ubiquitin-positive mapt-negative inclusions in frontotemporal lobar degeneration and amyotrophic lateral sclerosis [60].
6	optn	O	ALS-linked cellular aggregates, include FUS, TDP-43(TARDBP), and OPTN [61]
7	ang	O	1) Angiogenin promotes tumoral growth and angiogenesis, fus inhibitons repress angiogenesis [62]. 2) TDP-43 loss-of-function rescues the angiogenic defects [63].
8	grn	O	1) Grn affects tau phosphorylation [64]. 2) Grn mutations have abnormal accumulations of the TDP-43 protein in affected neurons [65].
9	taf15	O	TDP-43, FUS and TAF15 is associated with ALS and ALS-associated mutations identified in these genes are found in their C-terminal Gly-rich domains [66].

On the other hand, in the co-occurrence-based ABC model, 68 B entities were extracted and 15 B entities were evaluated as relevant genes with 22.1% precision. S2 Table shows the expert opinions and evidence on the B entities between FUS and TARDBP.

Table 10 shows the result of verifying B entities extracted by the context assignment based ABC model. Seven of the eight B entities were judged to be relevant with 87.5% precision.

**Table 10:** Verification of B entities from context-assignment based ABC model(FUS-TARDBP).

	B entity	Verifi- cation	Evidence
1	sod1	X	sod1 acts independently of fus and tardbp [49].
2	gli3	O	1) Fus is associated with ALS while, gli3 is associated with ALS through the shh pathway [50,51]. 2) Sonic hedgehog signaling in which gli3 participates and notch signaling can cooperate to regulate neurogenic divisions. Notch signaling may rescue tardbp [52,53].
3	c9orf72	O	1) Fus is associated with endosomal trafficking [54]. 2) Tardbp loss of function inhibits endosomal trafficking, c9orf72 regulates endosomal trafficking [55, 56].
4	vapb	O	1) Fus disrupts the vapb interactions to other signaling proteins [57]. 2) Mutations in vapb have already been shown to cause cytoplasmic transactive response tardbp accumulations [58].
5	mapt	O	1) Fus alternatively splices mapt [59]. 2) Tardbp is a component of ubiquitin-positive mapt-negative inclusions in frontotemporal lobar degeneration and amyotrophic lateral sclerosis [60].
6	optn	O	ALS-linked cellular aggregates, include FUS, TDP-43(TARDBP), and OPTN [61]
7	grn	O	1) Grn affects tau phosphorylation [64]. 2) Grn mutations have abnormal accumulations of the TDP-43 protein in affected neurons [65].
8	taf15	O	TDP-43, FUS and TAF15 is associated with ALS and ALS-associated mutations identified in these genes are found in their C-terminal Gly-rich domains [66].

## Discussion

### Extraction of biological context

It is difficult to extract both entity relations and biological contexts from a sentence. When an entity relation is extracted from a sentence, biological contexts may be not included. For this reason, only a small number of entity relations with biological context can be extracted. When applying biological context extraction, there are several points to consider.

First, the patterns of locations of the biological context have to be analyzed. In order to extract biological contexts, various patterns in which biological contexts are located should be considered. For example, in some abstracts, one sentence describes the biological context and the next sentence describes the entity relations related to the biological context in the previous sentence. Second, a solution to infer the biological context of entity relations which have no biological context is necessary. In this study, entity relations without context information were excluded in the context-based ABC model. The B entity from this model had high precision but low recall. In order to solve this problem, we proposed the context-assignment-based ABC model. The context-assignment-based ABC model showed similar performance to that of the context based ABC model. Therefore, in order to assign more precise biological context to an entity relation, a methodology for inferring biological context for each entity relation is required. Third, a more sophisticated analysis can be carried out assessing the importance of biological context elements. In this study, the importance of the biological context elements was not identified. The most important context element is organism; for example, the same medicine has a different effect in different organisms. Pharmaceutical companies sometimes fail to develop new drugs because experiments on human fail even after animal trials have proved a success. However, it is impossible to select the most important element among the other elements: cell, drug, and disease. In this study, the four biological context elements were assigned equal importance. However, if researchers want to extract specific knowledge, some elements may have greater importance than others. For example, if the ABC model is used to find gene interactions in certain diseases, the disease is a more important element than other elements. Therefore, if the importance of the context elements is identified, more sophisticated research can be carried out. All of the biological context elements in this study are independent. However, in fact all context elements have a relationship. For example, while there are diseases related to humans, there are diseases related to animals or plants. Prostate cancer has a relationship with prostate cells. There is also a relationship between each of biological context elements. Therefore, research into these relationships, will help to discover new hypothesis in bio-literature mining.

### Application of context based ABC model

Extracting entity relations with various biological contexts requires a generally well-known set of entity relations. In the context-based ABC model, the extracted entity relations are expressed as different entity relations depending on the biological context. For example, if the A–B entity relation has two different biological contexts, such as Alzheimer’s disease and Parkinson’s disease, this A–B entity relation is expressed as an A- B entity relation in Alzheimer’s disease and an A–B relation in Parkinson's disease. The proposed biological context extraction offers a new insight on hypothesis generation. For example, as shown in Table 11, the relation between APP and PSEN1 has nine biological contexts. The relation between APP and PSEN1 is well-known and was examined in at least nine studies. In the KEGG disease database, APP and PSEN1 are identified as representative genes for Alzheimer's disease. If entity relations have a small number of contexts, this may affect the generation of new hypotheses. For example, if an entity relation has only one biological context such as “Caenorhabditis elegans” or “Drosophila melanogaster”, it can serve as a base for a plausible hypothesis leading to new experiments. In addition, experiments about a specific entity relation can be carried out based on a new biological context such as mouse or human. The context-based ABC model can identify more accurate paths as the paths become longer. For example, in the co-occurrence-based ABC model, when an A-B-C-D path is constructed in a network, the path may not be relevant because of a lack of common biological conditions. Sometimes, a high frequency makes it possible to find a relevant short path.

**Table 11:** Example of context in relation APP-PSEN1.

Relation	Context	
	Name	Type
APP—PSEN1	myoclonus	DISEASE
	Lewy body dementia	DISEASE
	Dementia	DISEASE
	transgenic mouse	ORGANISM
	Tumor	DISEASE
	Alzheimer disease	DISEASE
	Melatonin	DRUG
	t-cell	CELL
	amyloid plaque	DISEASE

**Fig 7:**
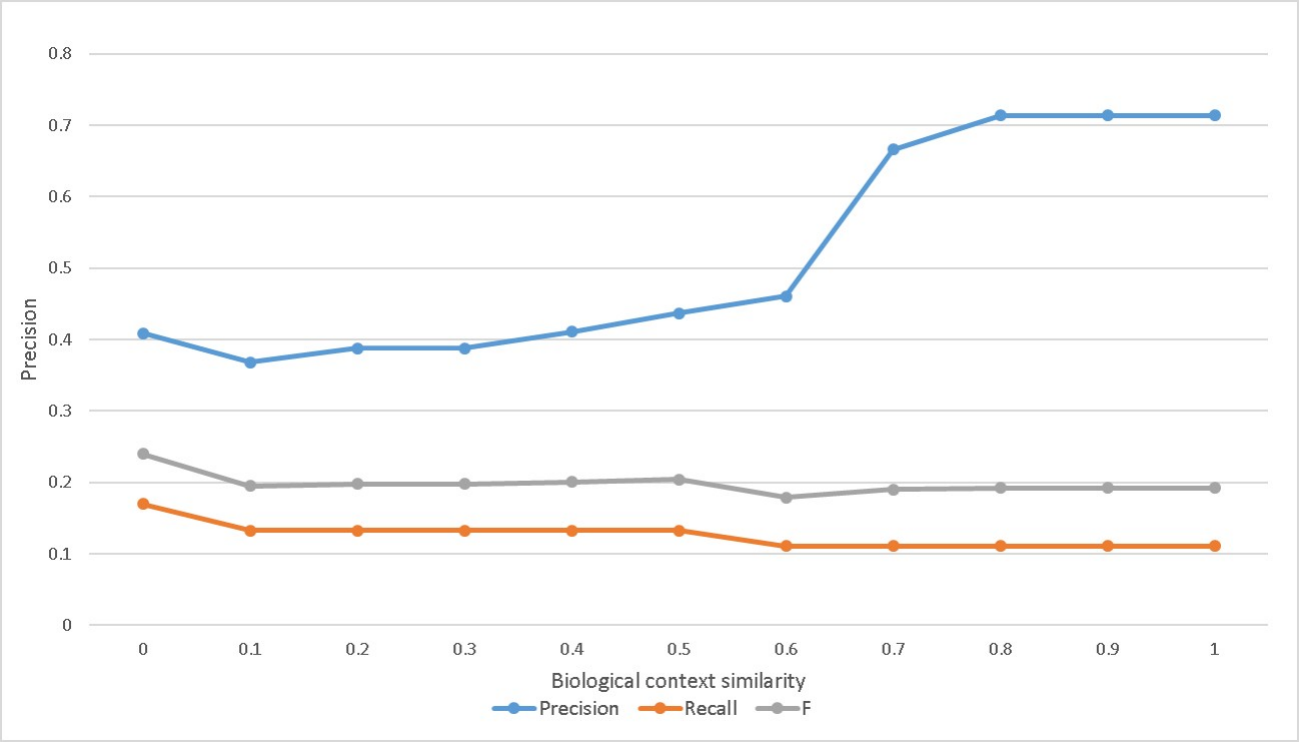
Precision, recall and F-measure of Context-based ABC model (APOE–MAPT).

However, frequency is limited in finding relevant long paths because frequency does not guarantee the consistency of biological conditions in the path. Therefore, in a long path, common mediation is required to ensure connection consistency. Common biological conditions can be framed in a biological context. In the context-based ABC model, the biological context of the initial entity relation of a path is the same as or similar to the biological context of the last entity relation. Therefore, it can be effective to conduct network-based analysis in large networks based on the context-based ABC model. The context-based ABC model filters the results of the co-occurrence-based ABC model. In this study, if the similarity between biological contexts was lower than the threshold, no connection between entity relations was constructed. These algorithms can reduce the number of false positives, which frequently occur in results of the co-occurrence-based ABC model. However, recall performance may deteriorate. Fig 7 shows the precision, recall, and F-measure of the context-based ABC model in our experiment, using the B entities of APOE and MAPT. It shows low recall and relatively high precision. And it also exhibits F value of 0.3 or less.

Although the context-based ABC model shows low recall, the application of the model was effective due to its high precision. In addition, the low recall of the context-based ABC model results from the discarding of entity relations without biological context. Therefore, if all entity relations can be extracted with their biological contexts, the performance of the model will be improved.

We propose the context-assignment-based ABC model as a solution to the recall problem. In this study, the context-assignment-based ABC model showed higher recall than the context-based ABC model in the case of “APOE–MAPT”. However, in the case of “FUS—TARDBP”, the results of the context-assignment-based ABC model had a lower recall value than those of the context-based ABC model. Because of the assignment of biological context to all entity relations from same abstract, two previously connected entity relations may be disconnected. If this weakness is overcome, results could be improved. Finally, it is difficult to verify experimental hypotheses using the ABC model. Expert intervention is essential to validate the ABC model's result. In previous studies, the validity of the study's results was evaluated through expert evaluation [24, 25, 29, 38, 39]. However, this method still has a limitation because it is subjective. In order to overcome these limitations, it is necessary to construct a corpus suitable for B entity or hypothesis verification in ABC model.

## Conclusions

The co-occurrence-based ABC model is one of the models that provides new hypotheses. An intermediate entity acts as a middleman between two other entity relations when applying the ABC model. However, due to the lack of a biological context, B entity extracted by the co-occurrence-based ABC model is sometimes not useful or relevant. In order to overcome the limitations of the co-occurrence-based ABC model, this study defined biological context, proposed a method to extract context from the literature, and then applied the biological contexts to ABC model. In this study the biological contexts are defined as cell, drug, disease, and organism; places where interactions take place in living organisms, or conditions which interfere with or promote such interactions. Using biological contexts, we propose the context-based ABC model, which provides more relevant B entities than the co-occurrence-based ABC model.

In addition to the context-based ABC model, we also proposed the context-assignment-based ABC model, which assigns biological context to entity relations using a combination of extracted biological contexts, and assigning them to all of the entity relations from the same abstract. The context-based ABC model provides a small number of B entities with high precision, although the recall performance may be lower than that of the co-occurrence-based ABC model, because entity relations without biological context are excluded when constructing paths. Therefore, a method of assigning the biological context to entity relations without biological context is required.

The context-assignment-based ABC model is our solution to this problem. In order to evaluate the performance of the context-based and the context-assignment ABC models, we verified the relevance of the B entities between well-known relations, APOE and MAPT, and FUS and TARDBP. The relevance of each B entity was verified by three experts, and each verification included the evidence for their decisions. The context-based ABC model showed a precision from 36.8% to 71.4% for APOE and MAPT, and FUS and TARDBP, showed a precision from 80% to 89%.

The context-assignment-based ABC model showed a precision of the relevance of the B entities between APOE and MAPT which rose from 42.8% to 70%, while the precision of the relevance of the B entities between FUS and TARDBP rose from 77.7% to 87.5%. On the other hand, the co-occurrence-based ABC model showed 27.1% precision for APOE and MAPT, and about 22.1% for FUS and TARDBP. The proposed methods provided more accurate paths than those of the co-occurrence-based ABC model. Therefore, biological context must be considered when the ABC model is applied. This study has the following limitations. First, we conducted an evaluation of our results in only two cases. As mentioned above, the ABC model is used to discover new knowledge or to create new hypotheses. It is difficult to find a validated corpus. Therefore, we used two closed-discovery cases to prove our results by experts. Second, verification of B entities is carried out manually. The manual evaluation has the limitation that it is subjective. However, in this study, in order to guarantee objectivity, evaluation was carried out by three domain experts. Although it is natural that the results are verified by experts, this procedure is limited by manual evaluation.

In the future, biological experiments should be performed to verify the effectiveness of the proposed models. The effectiveness of the context-based ABC model has been demonstrated through the verification of B entities between two well-known entities by experts using public databases and academic papers. However, this validation does not prove that the proposed methodology is valid for the generations of biological experiments. Thus, if we verify our proposed method through biological experiments, we can propose a more accurate hypothesis generation system.

## Supporting information

S1 Table Manual verification on B entities by expert (APOE—MAPT). (DOCX)

S2 Table Manual verification on B entities by expert (FUS—TARDBP). (DOCX)
