# Supplementary material for: A context-based ABC model for literature-based discovery
Source: PLoS One. 2019 Apr 24;14(4):e0215313. doi: 10.1371/journal.pone.0215313 (PMC6481912; doi:10.1371/journal.pone.0215313)
Supplement: S1 Table — (DOCX) [file pone.0215313.s001.docx]

Supporting Information

S1 Table. Manual verification on B entities by expert (APOE - MAPT)

| **Rank** | **B entity** | **Validity** | **Evidence** | **Expert**  **1** | **Expert**  **2** | **Expert**  **3** |
| --- | --- | --- | --- | --- | --- | --- |
| 1 | snca | X | 1) SNCA as well as ApoE has been associated with cognitive decline in neurodegenerative disease.  2) SNCA, mapt have no direct relation. | O | X | X |
| 2 | psen1 | X | ApoE, psen1, and mapt have no direct relation. | O | X | X |
| 3 | prnp | X | Mapt and prnp are in independent pathway having no relation. | X | X | X |
| 4 | app | O | 1) ApoE elevates the transcription of APP.  2) APP metabolism regulates mapt proteostasis. | O | O | O |
| 5 | clu | X | ApoE, clu, and mapt have no direct relation. | O | X | X |
| 6 | ywhaq | X | Mapt and ywhaq are in independent pathway having no relation | X | X | X |
| 7 | phgdh | O | 1) APOE and phgdh have interaction.  2) Serine (phgdh is involved in serine synthesis) mutations in mapt increases mapt aggregation. | O | O | O |
| 8 | sars2 | X | APOE and sars2 are in independent pathway having no relation | X | X | X |
| 9 | ins | O | 1) ApoE4 reduces brain insulin (ins).  2) Insulin dysfunction induces in vivo mapt hyperphosphorylation. | O | O | O |
| 10 | gfap | O | GFAP and ApoE is associated with increased phosphorylation of mapt | O | O | O |
| 11 | prkn | X | APOE and prkn are in independent pathway having no relation | X | X | X |
| 12 | psen2 | X | ApoE, psen2, and mapt have no direct relation. | O | X | X |
| 13 | cst3 | X | Mapt and CST3 are in independent pathway having no relation | X | X | X |
| 14 | artn | X | APOE and artn are in independent pathway having no relation | X | X | X |
| 15 | bche | O | 1) ApoE and Bche functions as modulators of cerebral amyloid deposition  2) BChE-K(BChE varient) is associated with reduced mapt phosphorylation. | O | O | O |
| 16 | serpina3 | X | APOE and serpina3 are in independent pathway having no relation | X | X | X |
| 17 | sorl1 | X | APOE and sorl1 are in independent pathway having no relation | X | X | X |
| 18 | grn | X | APOE and grn are in independent pathway having no relation | X | X | X |
| 19 | tnf | X | ApoE, tnf, and mapt have no direct relation. | O | X | X |
| 20 | bace1 | O | 1) APOE and BACE1 levels show relation  2) Mapt hyperphosphorylation is related with increased BACE1 | O | X | O |
| 21 | cdk5 | X | APOE, CDK5, and mapt are within the Alzheimer's disease pathway, but it is difficult to find relationship between ApoE and CDK5 and between CDK5 and mapt. | O | X | X |
| 22 | tardbp | O | 1) ApoE formed complex with TARDBP.  2) TARDBP and mapt protein levels are related. | O | O | O |
| 23 | picalm | O | 1) PICALM and APOE is associated.  2) PICALM modulates mapt accumulation. | O | X | O |
| 24 | sod1 | O | 1) Sod1 affects apoE expression.  2) Sod1 promotes mapt phosphorylation. | O | O | O |
| 25 | ca1 | X | 1) APOE and CA1 are in independent pathway having no relation.  2) Mapt and CA1 are in independent pathway having no relation. | X | X | X |
| 26 | htt | O | 1) ApoE polymorphism and 2 types of functional polymorphism in the 5-HTT gene have relation  2) The location of htt and mapt have relation. | O | X | O |
| 27 | ctsd | O | 1) CTSD and APOE have been associated with cognitive ability.  2) CTSD is associated with degrading mapt. | O | X | O |
| 28 | ngf | O | 1) NGF significantly increased ApoE.  2) NGF regultaes mapt. | O | O | O |
| 29 | mapk8 | X | ApoE and mapk8 are in independent pathway having no relation | X | X | X |
| 30 | lrrk2 | X | ApoE, lrrk2, and mapt have no direct relation. | O | X | X |
| 31 | mtor | O | 1) ApoE-deplition results in induced mtor.  2) Mtor and mapt expression are related. | O | O | O |
| 32 | jun | X | ApoE and JUN are in independent pathway having no relation . | X | X | X |
| 33 | ide | X | Mapt and IDE are in independent pathway having no relation. | X | X | X |
| 34 | bdnf | O | 1) ApoE and BDNF polymorphisms moderate amyloid β.  2) phosphorylation of mapt protein are regulated by BDNF. | O | O | O |
| 35 | nefl | X | ApoE and nefl are in independent pathway having no relation | X | X | X |
| 36 | sms | X | ApoE and SMS are in independent pathway having no relation | X | X | X |
| 37 | syp | X | Mapt and syp are in independent pathway having no relation | X | X | X |
| 38 | cyp46a1 | O | CYP46A1 may interact with APOE to influence phospho-mapt protein | O | O | O |
| 39 | tomm40 | X | ApoE, tomm40, and mapt have no direct relation. | O | X | X |
| 40 | c9orf72 | O | 1) c9orf72 is a stronger determinant than Apoe of cognitive impairment in ALS.  2) Mutations in c9orf72 and mapt are found in familial frontotemporal dementia. | O | O | O |
| 41 | chat | X | Mapt and chat are in independent pathway having no relation. | X | X | X |
| 42 | reln | O | Apolipoprotein E and Reln ligands modulate mapt. | O | O | O |
| 43 | il6 | X | APOE and il6 are in independent pathway having no relation. | X | X | X |
| 44 | mcidas | X | 1) ApoE is required for cell cycle regulation (mcidas plays a role in mitotic cell cycle progression by promoting cell cycle exit).  2) No direct evidence of interaction between mcidas and mapt. | X | O | X |
| 45 | bin1 | O | 1) BIN1 is a risk locus for Alzheimer's disease, after APOE.  2) BIN1 affects Alzheimer’s disease risk primarily by modulating mapt pathology, | O | O | O |
| 46 | ttr | X | Mapt and ttr are in independent pathway having no relation | X | X | X |
| 47 | vcp | X | APOE and vcp are in independent pathway having no relation | X | X | X |
| 48 | alb | X | ALB (Albumin) is in independent pathways of APOE and mapt. | X | X | X |
| 49 | cr1 | O | CR1 interacts with APOE and affects mapt. | O | O | O |
| 50 | ldlr | O | LDLR-immunoreactive inclusions localization is related with APOE and p-mapt. | O | O | O |
| 51 | duoxa1 | X | Mapt and duoxa1 are in independent pathway having no relation | X | X | X |
| 52 | slc26a4 | X | ApoE and slc26a4 are in independent pathway having no relation | X | X | X |
| 53 | hfe | O | 1) HFE mutations correlate with APOE.  2) HFE increases mapt phosphorylation. | O | O | O |
| 54 | il10 | O | IL-10 and ApoE suppress microglial Ab phagocytosis in vitro which changes mapt phosphorylation. | O | O | O |
| 55 | sirt2 | X | APOE and sirt2 are in independent pathway having no relation. | X | X | X |
| 56 | atxn3 | X | APOE and atxn3 are in independent pathway having no relation. | X | X | X |
| 57 | ca2 | X | APOE and CA2 are in independent pathway having no relation. | X | X | X |
| 58 | comt | X | Mapt and COMT are in independent pathway having no relation. | X | X | X |
| 59 | sth | X | Mapt and sth are in independent pathway having no relation. | X | X | X |
| 60 | a2m | X | ApoE, a2m, and mapt have no direct relation. | O | X | X |
| 61 | ncstn | X | Mapt and ncstn are in independent pathway having no relation. | X | X | X |
| 62 | rcan1 | O | ApoE genotype shows higher levels of RCAN1 and phospho-ta. | O | O | O |
| 63 | mapk1 | X | APOE, mapk1, and mapt are within the Alzheimer's disease pathway, but it is difficult to find relationship between ApoE and mapk1 and between mapk1 and mapt. | O | X | X |
| 64 | nms | X | APOE and nms are in independent pathway having no relation | X | X | X |
| 65 | ace | X | ApoE, ace, and mapt have no direct relation. | O | X | X |
| 66 | bcl2 | O | 1) APOE and bcl2 are involved in apoptosis.  2) Mapt dephosphorylation potentiates apoptosis by mechanisms involving a failed dephosphorylation/activation of Bcl-2. | O | O | O |
| 67 | sp1 | O | 1) SP1 regulates apoE gene  2) Sp1 partly co-localizes with hyper-phosphorylated mapt . | O | O | O |
| 68 | vegfa | O | 1) ApoE and vegfa levels are related.  2) Vegfa and mapt have a common regulator AKT. | O | X | O |
| 69 | cox8a | X | APOE and cox8a are in independent pathway having no relation | X | X | X |
| 70 | src | O | 1) ApoE binding stimulates intracellular activation of Src.  2) SRC family kinases phosphorylates mapt | O | O | O |
| 71 | tp53 | O | 1) Tp53 deletion and ApoE deltion accelerated aortic atherosclerosis.  2) p53 (Tp53 family) and mapt are both associated with neurodegenerative disorders | O | O | O |
| 72 | ccs | X | Mapt and ccs are in independent pathway having no relation | X | X | X |
| 73 | c3 | X | Mapt and c3 are in independent pathway having no relation | X | X | X |
| 74 | fasn | X | APOE and fasn are in independent pathway having no relation | X | X | X |
| 75 | timm8a | X | Mapt and timm8a are in independent pathway having no relation | X | X | X |
| 76 | ceruloplasmin | O | 1) Ceruloplasmin/transferrin ratio, and the APOE is associated.  2) Ceruloplasmin and mapt phosphorylation is associated. | O | X | O |
| 77 | lrp1 | O | Mapt and LRP1 might modify the risk of Alzheimer’s disease in an APOE manner. | O | O | O |
| 78 | s100b | O | 1) Release of S-100B is associated with APOE.  2) S100B induces mapt protein hyperphosphorylation. | O | O | O |
| 79 | tff1 | X | APOE and TFF1 are in independent pathway having no relation. | X | X | X |
| 80 | dlg4 | X | APOE and dlg4 are in independent pathway having no relation. | X | X | X |
| 81 | apob | X | ApoE, apob, and mapt have no direct relation. | O | X | X |
| 82 | mme | X | APOE and mme are in independent pathway having no relation | X | X | X |
| 83 | pold1 | X | mapt and pold1 are in independent pathway having no relation | X | X | X |
| 84 | eaf2 | X | APOE and eaf2 are in independent pathway having no relation | X | X | X |
| 85 | nos3 | X | APOE and nos3 are in independent pathway having no relation | X | X | X |
| 86 | serpina2 | X | APOE and serpina2 are in independent pathway having no relation | X | X | X |
| 87 | trem2 | O | 1) TREM2 binds to APOE.  2) Silencing of trem2 exacerbates mapt pathology. | O | O | O |
| 88 | abca7 | O | 1) ABCA7, and APOE genes affect the features of cognitive impairment.  2) ABCA7 was associated with paired helical filament mapt tangle density. | O | X | O |
| 89 | dapk1 | X | APOE and dapk1 are in independent pathway having no relation. | X | X | X |
| 90 | eif2ak2 | X | APOE and eif2ak2 are in independent pathway having no relation. | X | X | X |
| 91 | sncaip | X | APOE and sncaip are in independent pathway having no relation. | X | X | X |
| 92 | ctsb | X | APOE and ctsb are in independent pathway having no relation. | X | X | X |
| 93 | psenen | X | Mapt and psenen are in independent pathway having no relation. | X | X | X |
| 94 | syt1 | X | Mapt and syt1 are in independent pathway having no relation. | X | X | X |
| 95 | tcof1 | X | APOE and TCOF1 are in independent pathway having no relation. | X | X | X |
| 96 | bank1 | x | APOE and BANK1 are in independent pathway having no relation | x | x | x |
| 97 | bap1 | X | APOE and bap1 are in independent pathway having no relation | X | X | X |
| 98 | casp3 | X | ApoE, CASP3, and mapt are within the Alzheimer's disease pathway, but it is difficult to find relationship between ApoE and casp3 and between casp3 and mapt. | O | X | X |
| 99 | crp | O | 1) ApoE gene variations and plasma levels of CRP are associated.  2) CRP contributes to the releasing of mapt. | O | O | O |
| 100 | ctnnbl1 | X | Mapt and ctnnbl1 are in independent pathway having no relation. | X | X | X |
| 101 | fos | X | APOE and fos are in independent pathway having no relation. | X | X | X |
| 102 | mos | X | APOE and mos are in independent pathway having no relation. | X | X | X |
| 103 | rhoa | X | Mapt and rhoa are in independent pathway having no relation | X | X | X |
| 104 | tnfrsf11a | X | APOE and TNFRSF11A are in independent pathway having no relation | X | X | X |
| 105 | uchl1 | X | Mapt and uchl1 are in independent pathway having no relation | X | X | X |
| 106 | hspg2 | X | ApoE, hspg2, and mapt have no direct relation. | O | X | X |
| 107 | map2 | X | ApoE, map2, and mapt have no direct relation. | O | X | X |
| 108 | npy | X | Mapt and npy are in independent pathway having no relation. | X | X | X |
| 109 | plg | X | Mapt and plg are in independent pathway having no relation. | X | X | X |
| 110 | rab7a | X | APOE and rab7a are in independent pathway having no relation. | X | X | X |
| 111 | slc6a4 | X | APOE and slc6a4 are in independent pathway having no relation. | X | X | X |
| 112 | tnfsf10 | X | Mapt and tnfsf10 are in independent pathway having no relation. | X | X | X |
| 113 | aph1b | X | Mapt and aph1b are in independent pathway having no relation. | X | X | X |
| 114 | ccr2 | X | ApoE, ccr2, and mapt have no direct relation. | O | X | X |
| 115 | cdipt | X | APOE and cdipt are in independent pathway having no relation. | X | X | X |
| 116 | champ1 | X | APOE and champ1 are in independent pathway having no relation. | X | X | X |
| 117 | epo | X | Mapt and epo are in independent pathway having no relation. | X | X | X |
| 118 | jag1 | X | mapt and jag1 are in independent pathway having no relation | X | X | X |
| 119 | nps | X | APOE and nps are in independent pathway having no relation. | X | X | X |
| 120 | spin1 | X | Mapt and spin1 are in independent pathway having no relation. | X | X | X |
| 121 | tnfaip1 | X | APOE and TNFAIP1 are in independent pathway having no relation. | X | X | X |
| 122 | acss2 | X | APOE and acss2 are in independent pathway having no relation. | X | X | X |
| 123 | aph1a | X | APOE and aph1a are in independent pathway having no relation. | X | X | X |
| 124 | bace2 | O | 1) APOE and BACE2 levels show relation  2) Mapt hyperphosphorylation is related with increased BACE2. | O | X | O |
| 125 | cd40lg | X | 1)APOE and cd40lg are in independent pathway having no relation | X | X | X |
| 126 | ch25h | O | ApoE and CH25H affect mapt phosphorylation . | O | X | O |
| 127 | coil | X | ApoE and COIL are in independent pathway having no relation. | X | X | X |
| 128 | il1b | X | ApoE, il1b, and mapt have no direct relation. | O | X | X |
| 129 | pltp | O | 1) PLTP interacts with apoE.  2) PLTP-induced reduction in mapt phosphorylation. | O | O | O |
| 130 | ppp1r12a | X | Mapt and ppp1r12a are in independent pathway having no relation. | X | X | X |
| 131 | uhmk1 | X | APOE and uhmk1 are in independent pathway having no relation. | X | X | X |
| 132 | agrn | X | APOE and agrn are in independent pathway having no relation. | X | X | X |
| 133 | bsg | X | Mapt and bsg are in independent pathway having no relation. | X | X | X |
| 134 | c1s | X | APOE and c1s are in independent pathway having no relation. | X | X | X |
| 135 | ca4 | X | APOE and CA4 are in independent pathway having no relation. | X | X | X |
| 136 | cd40 | X | APOE and cd40 are in independent pathway having no relation. | X | X | X |
| 137 | cx3cr1 | O | 1) CX3CR1 deficiency promotes muscle repair and regeneration by enhancing macrophage ApoE production.  2) CX3CR1 aggravates mapt pathology | O | X | O |
| 138 | cxcl12 | O | 1) Apoe-Deficient affects cxcl12.  2) CXCL12 plasma levels significantly inversely correlated with mapt. | O | O | O |
| 139 | hcrt | X | APOE and hcrt are in independent pathway having no relation. | X | X | X |
| 140 | kng1 | x | APOE and kng1 are in independent pathway having no relation. | x | x | x |
| 141 | lat2 | X | Mapt and lat2 are in independent pathway having no relation. | X | X | X |
| 142 | mobp | X | APOE and mobp are in independent pathway having no relation. | X | X | X |
| 143 | mok | X | APOE and mok are in independent pathway having no relation. | X | X | X |
| 144 | nfe2l2 | O | Nfe2l2 affects apoe expression and mapt phosphorylation. | O | O | O |
| 145 | pgap1 | X | APOE and pgap1 are in independent pathway having no relation. | X | X | X |
| 146 | slc3a2 | X | APOE and slc3a2 are in independent pathway having no relation. | X | X | X |
| 147 | son | X | Mapt and son are in independent pathway having no relation. | X | X | X |
| 148 | stx6 | X | Mapt and stx6 are in independent pathway having no relation. | X | X | X |
| 149 | c1orf56 | X | APOE and c1orf56 are in independent pathway having no relation. | X | X | X |
| 150 | cck | X | Mapt and cck are in independent pathway having no relation. | X | X | X |
| 151 | cd200 | O | APOE, CD200, and mapt shows impact on each other. | O | X | O |
| 152 | celf1 | X | APOE and celf1 are in independent pathway having no relation. | X | X | X |
| 153 | chgb | X | APOE and chgb are in independent pathway having no relation. | X | X | X |
| 154 | chrnb2 | X | Mapt and chrnb are in independent pathway having no relation. | X | X | X |
| 155 | crhr1 | X | APOE and crhr1 are in independent pathway having no relation. | X | X | X |
| 156 | eif2ak3 | O | 1) APOE affects EIF2AK3 association.  2) EIF2AK3 is related to mapt expression. | O | X | O |
| 157 | fermt2 | X | APOE and fermt2 are in independent pathway having no relation. | X | X | X |
| 158 | igf1 | O | Deletion of igf-1 effected mapt phosphorylation and these effects was stronger in impaired ApoE condition. | O | O | O |
| 159 | lxn | X | APOE and LXN are in independent pathway having no relation. | X | X | X |
| 160 | ms | X | APOE and ms are in independent pathway having no relation. | X | X | X |
| 161 | nos1 | O | ApoE, nos1, and mapt have no direct relation. | O | X | X |
| 162 | nova2 | X | APOE and nova2 are in independent pathway having no relation. | X | X | X |
| 163 | park16 | X | APOE and park16 are in independent pathway having no relation. | X | X | X |
| 164 | parp1 | X | APOE and parp1 are in independent pathway having no relation. | X | X | X |
| 165 | rps6kb1 | X | Mapt and rps6kb1 are in independent pathway having no relation. | X | X | X |
| 166 | tlr9 | O | 1) TLR9 has a role in the development of atherosclerosis in apolipoprotein E-deficient conditions.  2) TLR stimulation can promote an increase in mapt pathology. | O | O | O |
