# Supplementary material for: A context-based ABC model for literature-based discovery
Source: PLoS One. 2019 Apr 24;14(4):e0215313. doi: 10.1371/journal.pone.0215313 (PMC6481912; doi:10.1371/journal.pone.0215313)
Supplement: S2 Table — (DOCX) [file pone.0215313.s002.docx]

Supporting Information

S2 Table. Manual verification on B entities by expert (FUS - TARDBP)

| **Rank** | **B entity** | **Validity** | **Evidence** | **Expert**  **1** | **Expert**  **2** | **Expert**  **3** |
| --- | --- | --- | --- | --- | --- | --- |
| 1 | unc13a | O | 1) UNC-13/UNC13A in regulating motor neuron degeneration.  2) Mutant TDP-43 or FUS in their motor neurons show age-dependent motility defects leading to paralysis and motor neuron degeneration. | O | X | O |
| 2 | nefh | X | Nefh and fus are in independent pathways having no relation. | X | X | X |
| 3 | app | X | App and fus are in independent pathways having no relation. | X | X | X |
| 4 | rtn4 | X | Tardbp, FUS, and Rtn4 have no direct relation. | O | X | X |
| 5 | fbn1 | X | Fbn1 and fus are in independent pathways having no relation. | X | X | X |
| 6 | setx | X | Setx and tardp are in independent pathways having no relation. | X | X | X |
| 7 | sod1 | X | Sod1 acts independently of fus and tardbp. | X | X | X |
| 8 | sqstm1 | X | Sqstm1, FUS, and tardbp have no direct relation. | O | X | X |
| 9 | ubqln2 | O | UBQLN2, present inclusions co-localized with TDP-43 and FUS which shows high possibility of interaction(direct or indirect). | O | O | O |
| 10 | lat2 | X | Lat2 and tardp are in independent pathways having no relation. | X | X | X |
| 11 | vcp | O | 1) VCP has also been shown to interact genetically with FUS.  2) VCP and TDP-43 interact genetically and disease-causing mutations in VCP lead to redistribution of TDP-43. | O | O | O |
| 12 | psen2 | X | Fus, psen2, and tardbp have no direct relation | O | X | X |
| 13 | tars | X | Tars and fus are in independent pathways having no relation. | X | X | X |
| 14 | grn | O | 1) GRN affects tau phosphorylation.  2) GRN mutations have abnormal accumulations of the TDP-43 protein in affected neurons. | O | O | O |
| 15 | hnrnpa2b1 | O | 1) Fus contained hnRNP A2/B1 binding sites.  2) TDP-43 suppresses CGG repeat-induced neurotoxicity through interactions with HnRNP A2/B1. | O | O | O |
| 16 | prnp | X | Pnrp and fus are in independent pathways having no relation. | X | X | X |
| 17 | g3bp1 | O | 1) TDP-43 and tardbp interaction may affect G3BP1 exprssion.  2) G3BP1 is a target of TDP-43. | O | O | O |
| 18 | lrrk2 | X | :rrk2 and tardp are in independent pathways having no relation. | X | X | X |
| 19 | chmp2b | X | TARDBP, VCP, FUS, and CHMP2B have no direct relation. | O | X | X |
| 20 | optn | O | ALS-linked cellular aggregates, include FUS, TDP-43(TARDBP), and OPTN. | O | O | O |
| 21 | mapt | O | 1) FUS alternatively splices Mapt (Tau).  2) TARDBP is a component of ubiquitin-positive mapt-negative inclusions in frontotemporal lobar degeneration and amyotrophic lateral sclerosis. | O | O | O |
| 22 | atxn2 | O | 1) ATXN2 and FUS accumulation is associated with ALS.  2) ATXN2's interaction with TDP-43. | O | X | O |
| 23 | spg11 | X | Spg11 and tardp are in independent pathways having no relation. | X | X | X |
| 24 | matr3 | O | 1) FUS and MATR3 are both RNA binding proteins and participates in motor neuron degenerative disease.  2) MATR3 is an RNA- and DNA-binding protein that interacts with TDP-43. | O | O | O |
| 25 | trem2 | X | Trem2 and fus are in independent pathways having no relation. | X | X | X |
| 26 | fig4 | X | TARDP43, FUS, and FIG4 have no direct relation. | O | X | X |
| 27 | gli3 | O | 1) Fus is associated with ALS while, gli3 is associated with ALS through the shh pathway.  2) Sonic hedgehog signaling in which gli3 participates and notch signaling can cooperate to regulate neurogenic divisions. Notch signaling may rescue tardbp phenotype. | O | O | O |
| 28 | hfe | X | Hfe and fus are in independent pathways having no relation. | X | X | X |
| 29 | csf1r | x | Csf1r and tardp are in independent pathways having no relation. | x | x | x |
| 30 | rbms3 | X | Rbms3 and fus are in independent pathways having no relation. | X | X | X |
| 31 | pink1 | X | Pink1 and tardp are in independent pathways having no relation. | X | X | X |
| 32 | htt | X | Htt and fus are in independent pathways having no relation. | X | X | X |
| 33 | sigmar1 | X | Sigmar1 and tardp are in independent pathways having no relation. | X | X | X |
| 34 | als2 | X | Als2 and tardp are in independent pathways having no relation. | X | X | X |
| 35 | ang | O | 1) Angiogenin promotes tumoral growth and angiogenesis, fus inhibitons repress angiogenesis.  2) TDP-43 loss-of-function rescues the angiogenic defects. | O | O | O |
| 36 | ccnf | X | TARDP43, FUS, and CCNF have no direct relation. | O | X | X |
| 37 | dctn1 | X | Dctn and tardp are in independent pathways having no relation. | X | X | X |
| 38 | taf15 | O | TDP-43, FUS and TAF15 is associated with ALS and ALS-associated mutations identified in these genes are found in their C-terminal Gly-rich domains. | O | O | O |
| 39 | ewsr1 | X | TARDP43, FUS, and EWSR1 have no direct relation. | O | X | X |
| 40 | tuba4a | X | Tuba4a and tardp are in independent pathways having no relation. | X | X | X |
| 41 | vapb | O | 1) FUS disrupts the VAPB interactions to other signlaing porteins.  2) Mutations in VAPB have already been shown to cause cytoplasmic transactive response TARDBP accumulations. | O | O | O |
| 42 | chrna3 | X | Chrna3 and tardp are in independent pathways having no relation. | X | X | X |
| 43 | ywhaq | X | Ywhaq and tardp are in independent pathways having no relation. | X | X | X |
| 44 | rnase4 | X | Rnase4 and tardp are in independent pathways having no relation. | X | X | X |
| 45 | vegfa | X | Vegfa and fus are in independent pathways having no relation. | X | X | X |
| 46 | gmnn | X | Gmnn and fus are in independent pathways having no relation. | X | X | X |
| 47 | c9orf72 | O | 1) Fus is associated with endosomal trafficking.  2) Tardbp loss of function inhibits endosomal trafficking, c9orf72 regulates endosomal trafficking. | O | O | O |
| 48 | malat1 | X | Malat1 and tardp are in independent pathways having no relation. | X | X | X |
| 49 | hnrnpa1 | X | Hnrnpa1 and tardp are in independent pathways having no relation. | X | X | X |
| 50 | mfn2 | X | Mfn2 and tardp are in independent pathways having no relation. | X | X | X |
| 51 | psen1 | X | Psen1 and fus are in independent pathways having no relation. | X | X | X |
| 52 | atxn1 | X | Atxn1 and tardp are in independent pathways having no relation. | X | X | X |
| 53 | elavl1 | X | Elavl1 and fus are in independent pathways having no relation. | X | X | X |
| 54 | mos | X | Mos and fus are in independent pathways having no relation. | X | X | X |
| 55 | chrna4 | X | Chrna4 and fus are in independent pathways having no relation. | X | X | X |
| 56 | ss18l1 | X | Ss18l1 and tardp are in independent pathways having no relation. | X | X | X |
| 57 | pfn1 | X | TARDP43, FUS, and PFN1 have no direct relation | O | X | X |
| 58 | phgdh | X | Phgdh and tardp are in independent pathways having no relation. | X | X | X |
| 59 | smn1 | X | TARDP43, FUS, and SMN1 have no direct relation. | O | X | X |
| 60 | stmn1 | X | Stmn1 and tardp are in independent pathways having no relation. | X | X | X |
| 61 | artn | X | Artn and tardp are in independent pathways having no relation. | X | X | X |
| 62 | tbk1 | X | TARDP43, FUS, and tbk1 have no direct relation. | O | X | X |
| 63 | tnpo1 | X | Tnpo1 and tardp are in independent pathways having no relation. | X | X | X |
| 64 | fate1 | X | Fate1 and fus are in independent pathways having no relation. | X | X | X |
| 65 | chchd10 | X | Chchd10 and tardp are in independent pathways having no relation. | X | X | X |
| 66 | chrnb4 | X | Chrnb4 and tardp are in independent pathways having no relation. | X | X | X |
| 67 | snord87 | X | Snord87 and tardp are in independent pathways having no relation. | X | X | X |
| 68 | nipa1 | X | Tardp43, FUS, and NIPA1 have no direct relation. | O | X | X |
